# Bis(2,6-diamino-4-chloro­pyrimidin-1-ium) fumarate

**DOI:** 10.1107/S1600536812045308

**Published:** 2012-11-10

**Authors:** Kaliyaperumal Thanigaimani, Nuridayanti Che Khalib, Abbas Farhadikoutenaei, Suhana Arshad, Ibrahim Abdul Razak

**Affiliations:** a School of Physics, Universiti Sains Malaysia, 11800 USM, Penang, Malaysia

## Abstract

In the title salt, 2C_4_H_6_ClN_4_^+^·C_4_H_2_O_4_^2−^, the complete fumarate dianion is generated by crystallographic inversion symmetry. The cation is essentially planar, with a maximum deviation of 0.018 (1) Å. In the anion, the carboxyl­ate group is twisted slightly away from the attached plane, the dihedral angle between the carboxyl­ate and (*E*)-but-2-ene planes being 12.78 (13)°. In the crystal, the protonated N atom and the 2-amino group of the cation are hydrogen bonded to the carboxyl­ate O atoms of the anion *via* a pair of N—H⋯O hydrogen bonds, forming an *R*_2_^2^(8) ring motif. In addition, another type of *R*_2_^2^(8) motif is formed by centrosymmetrically related pyrimidinium cations *via* N—H⋯N hydrogen bonds. These two combined motifs form a heterotetra­mer. The crystal structure is further stabilized by stong N—H⋯O, N—H⋯Cl and weak C—H⋯O hydrogen bonds, resulting a three-dimensional network.

## Related literature

For applications of pyrimidine derivatives, see: Condon *et al.* (1993 ▶); Maeno *et al.* (1990 ▶); Gilchrist (1997 ▶). For details of fumaric acid, see: Batchelor *et al.* (2000 ▶). For hydrogen-bonded synthons, see: Thakur & Desiraju (2008 ▶). For hydrogen-bond motifs, see: Bernstein *et al.* (1995 ▶). For bond-length data, see: Allen *et al.* (1987 ▶). For stability of the temperature controller used for the data collection, see: Cosier & Glazer (1986 ▶). 
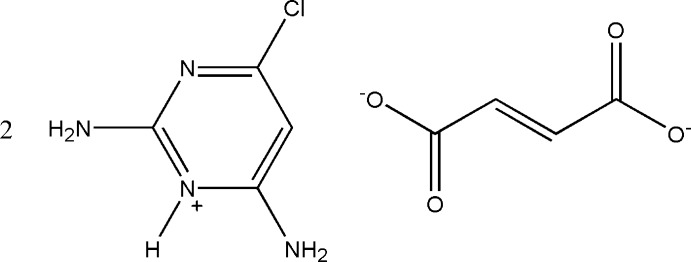


## Experimental

### 

#### Crystal data

C_4_H_6_ClN_4_^+^·0.5C_4_H_2_O_4_^2−^
*M* *_r_* = 202.61
Monoclinic, 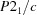

*a* = 5.4478 (7) Å
*b* = 10.5187 (14) Å
*c* = 14.8171 (18) Å
β = 100.990 (4)°
*V* = 833.50 (18) Å^3^
*Z* = 4
Mo *K*α radiation
μ = 0.43 mm^−1^
*T* = 100 K
0.71 × 0.31 × 0.17 mm

#### Data collection

Bruker SMART APEXII DUO CCD area-detector diffractometer
Absorption correction: multi-scan (*SADABS*; Bruker, 2009 ▶) *T*_min_ = 0.749, *T*_max_ = 0.931
9206 measured reflections
2984 independent reflections
2708 reflections with *I* > 2σ(*I*)
*R*_int_ = 0.033

#### Refinement

*R*[*F* ^2^ > 2σ(*F* ^2^)] = 0.042
*wR*(*F* ^2^) = 0.127
*S* = 1.08
2984 reflections
138 parameters
1 restraint
H atoms treated by a mixture of independent and constrained refinement
Δρ_max_ = 0.78 e Å^−3^
Δρ_min_ = −0.78 e Å^−3^

### 

Data collection: *APEX2* (Bruker, 2009 ▶); cell refinement: *SAINT* (Bruker, 2009 ▶); data reduction: *SAINT*; program(s) used to solve structure: *SHELXTL* (Sheldrick, 2008 ▶); program(s) used to refine structure: *SHELXTL*; molecular graphics: *SHELXTL*; software used to prepare material for publication: *SHELXTL* and *PLATON* (Spek, 2009 ▶).

## Supplementary Material

Crystal structure: contains datablock(s) global, I. DOI: 10.1107/S1600536812045308/rz5019sup1.cif

Structure factors: contains datablock(s) I. DOI: 10.1107/S1600536812045308/rz5019Isup2.hkl

Supplementary material file. DOI: 10.1107/S1600536812045308/rz5019Isup3.cml

Additional supplementary materials:  crystallographic information; 3D view; checkCIF report

## Figures and Tables

**Table 1:** Hydrogen-bond geometry (Å, °)

*D*—H⋯*A*	*D*—H	H⋯*A*	*D*⋯*A*	*D*—H⋯*A*
N2—H1⋯O2^i^	0.86 (1)	1.69 (1)	2.5281 (14)	165 (3)
N3—H2⋯O1^i^	0.81 (2)	2.12 (2)	2.9233 (15)	168 (2)
N3—H3⋯N1^ii^	0.85 (2)	2.15 (2)	3.0014 (16)	176 (2)
N4—H4⋯O1^iii^	0.78 (2)	2.08 (2)	2.8307 (16)	161 (2)
N4—H5⋯Cl1^iv^	0.77 (2)	2.78 (2)	3.3671 (13)	135.0 (19)
N4—H5⋯O2^i^	0.77 (2)	2.56 (2)	3.1458 (15)	134.2 (19)
C3—H3*A*⋯O2^v^	0.95	2.39	3.3085 (16)	162

Symmetry codes: (i) 
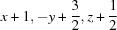
; (ii) 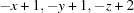; (iii) 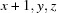; (iv) 
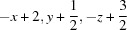
; (v) 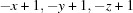.

**Fig. 1.**
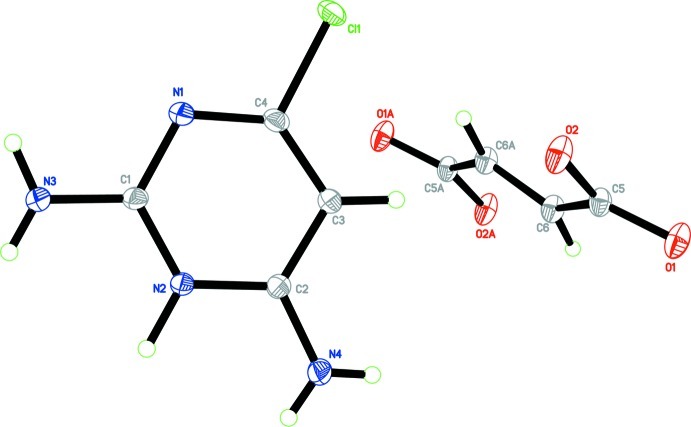
The molecular structure of the title compound with 50% probability displacement ellipsoids.

**Fig. 2.**
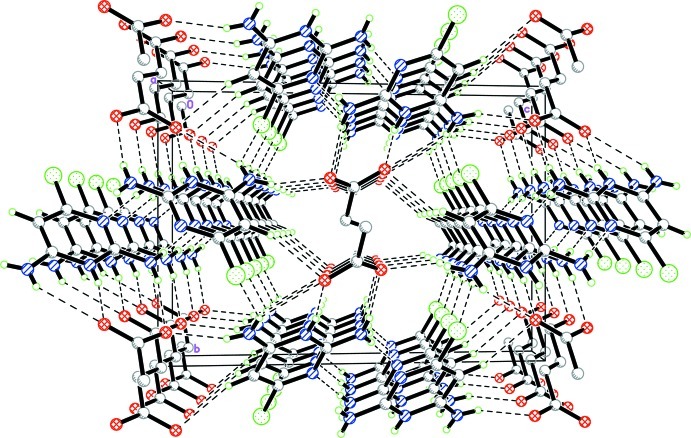
The crystal packing of the title compound viewed down the a axis. Hydrogen atoms not involved in the intermolecular interactions (dashed lines) have been omitted for clarity.

## supplementary crystallographic information

### Comment

Pyrimidine derivatives are very important molecules in biology and have many application in the areas of pesticide and pharmaceutical agents (Condon *et al.*, 1993). For example, imazosulfuron, ethirmol and mepanipyrim have been commercialized as agrochemicals (Maeno *et al.*, 1990). Pyrimidine derivatives have also been developed as antiviral agents, such as AZT, which is the most widely-used anti-AIDS drug (Gilchrist, 1997). Fumaric acid is among the organic compounds widely found in nature, and is a key intermediate in the biosynthesis of organic acids. Fumaric acid is of interest since it is known to form supramolecular assemblies with *N*-aromatic complexes (Batchelor *et al.*, 2000). In order to study some interesting hydrogen bonding interactions, the synthesis and structure of the title compound is presented here.

The asymmetric unit of title compound (Fig. 1), consists of a 2,6-diamino-4-chloropyrimidinium cation and a half of a fumarate dianion where the complete fumarate dianion is generated by crystallographic inversion symmetry (-*x* + 1, -*y* + 1, -*z* + 1). In the 2,6-diamino-4-chloropyridinium cation, protonatation of N1 atom has lead to a slight increase in the C1—N2—C2 angle (120.34 (10)°). The 2,6-diamino-4-chloropyridinium cation is essentially planar, with a maximum deviation of 0.018 (1) Å for atom C3. In the fumarate dianion, C5/C6/C5A/C6A plane makes a dihedral angle of 81.89 (6)° with 2,6-diamino-4-chloropyridinium cation. In the anion, the carboxylate group is twisted slightly away from the attached plane; the dihedral angle between the C5/C6/C5A/C6A and O1/O2/C5/C6 planes is 12.78 (13)°. The bond lengths (Allen *et al.*, 1987) and angles are normal.

In the crystal structure (Fig. 2), the protonated N atom and the 2-amino group of the cation are hydrogen bonded to the carboxylate O atoms of the anion *via* a pair of N—H···O hydrogen bonds, forming *R*_2_^2^(8) (Bernstein *et al.*, 1995) ring motifs. In addition, another type of *R*_2_^2^(8) motif is formed by centrosymmetrically related pyrimidinium cation through a pair of N3—H3···N1^iii^ hydrogen bonds (symmetry codes in Table 1). These two different motifs generate a linear heterotetrameric unit known to be one of the most stable synthons (Thakur & Desiraju, 2008). One of the O atoms of the carboxylate group acts as an acceptors of bifurcated N2—H1···O2^ii^ and N4—H5···O2^ii^ hydrogen bonds (symmetry codes in Table 1). The crystal structure is further stabilized by strong N4—H4···O1^iv^, N4—H5···Cl1^V^ and weak C3—H3A···O2^i^ hydrogen bonds (symmetry codes in Table 1), resulting in a three-dimensional network.

### Experimental

Hot methanol solutions (20 ml) of 2,6-diamino-4-chloropyrimidine (36 mg, Aldrich) and fumaric acid (29 mg, Merck) were mixed and warmed over a heating magnetic stirrer hotplate for a few minutes. The resulting solution was allowed to cool slowly at room temperature and crystals of the title compound appeared after a few days.

### Refinement

N-bound H Atoms were located in a difference Fourier maps and refined isotropically. The N2–H1 bond length was constrained to 0.85 (1) Å. The remaining hydrogen atoms were positioned geometrically [C–H= 0.95 Å] and were refined using a riding model, with *U*_iso_(H) = 1.2 *U*_eq_(C).

### Crystal data

**Table:**

C_4_H_6_ClN_4_^+^·0.5C_4_H_2_O_4_^2^^−^	*F*(000) = 416
*M**_r_* = 202.61	*D*_x_ = 1.615 Mg m^−^^3^
Monoclinic, *P*2_1_/*c*	Mo *K*α radiation, λ = 0.71073 Å
Hall symbol: -P 2ybc	Cell parameters from 6512 reflections
*a* = 5.4478 (7) Å	θ = 3.4–32.6°
*b* = 10.5187 (14) Å	µ = 0.43 mm^−^^1^
*c* = 14.8171 (18) Å	*T* = 100 K
β = 100.990 (4)°	Block, colourless
*V* = 833.50 (18) Å^3^	0.71 × 0.31 × 0.17 mm
*Z* = 4	

### Data collection

**Table:**

Bruker SMART APEXII DUO CCD area-detector diffractometer	2984 independent reflections
Radiation source: fine-focus sealed tube	2708 reflections with *I* > 2σ(*I*)
Graphite monochromator	*R*_int_ = 0.033
φ and ω scans	θ_max_ = 32.6°, θ_min_ = 2.4°
Absorption correction: multi-scan (*SADABS*; Bruker, 2009)	*h* = −8→8
*T*_min_ = 0.749, *T*_max_ = 0.931	*k* = −15→11
9206 measured reflections	*l* = −22→20

### Refinement

**Table:**

Refinement on *F*^2^	Primary atom site location: structure-invariant direct methods
Least-squares matrix: full	Secondary atom site location: difference Fourier map
*R*[*F*^2^ > 2σ(*F*^2^)] = 0.042	Hydrogen site location: inferred from neighbouring sites
*wR*(*F*^2^) = 0.127	H atoms treated by a mixture of independent and constrained refinement
*S* = 1.08	*w* = 1/[σ^2^(*F*_o_^2^) + (0.0781*P*)^2^ + 0.3432*P*] where *P* = (*F*_o_^2^ + 2*F*_c_^2^)/3
2984 reflections	(Δ/σ)_max_ < 0.001
138 parameters	Δρ_max_ = 0.78 e Å^−^^3^
1 restraint	Δρ_min_ = −0.78 e Å^−^^3^

### Special details

**Table:**

Experimental. The crystal was placed in the cold stream of an Oxford Cryosystems Cobra open-flow nitrogen cryostat (Cosier & Glazer, 1986) operating at 100.0 (1) K.
Geometry. All e.s.d.'s (except the e.s.d. in the dihedral angle between two l.s. planes) are estimated using the full covariance matrix. The cell e.s.d.'s are taken into account individually in the estimation of e.s.d.'s in distances, angles and torsion angles; correlations between e.s.d.'s in cell parameters are only used when they are defined by crystal symmetry. An approximate (isotropic) treatment of cell e.s.d.'s is used for estimating e.s.d.'s involving l.s. planes.
Refinement. Refinement of *F*^2^ against ALL reflections. The weighted *R*-factor *wR* and goodness of fit *S* are based on *F*^2^, conventional *R*-factors *R* are based on *F*, with *F* set to zero for negative *F*^2^. The threshold expression of *F*^2^ > σ(*F*^2^) is used only for calculating *R*-factors(gt) *etc*. and is not relevant to the choice of reflections for refinement. *R*-factors based on *F*^2^ are statistically about twice as large as those based on *F*, and *R*- factors based on ALL data will be even larger.

### Fractional atomic coordinates and isotropic or equivalent isotropic displacement parameters (Å^2^)

**Table:**

	*x*	*y*	*z*	*U*_iso_*/*U*_eq_	
Cl1	0.40235 (6)	0.33920 (3)	0.76706 (2)	0.01926 (11)	
N1	0.60987 (19)	0.49304 (10)	0.89510 (7)	0.0145 (2)	
N2	0.94266 (19)	0.63383 (10)	0.88172 (7)	0.01248 (19)	
N3	0.7774 (2)	0.62835 (11)	1.01344 (8)	0.0172 (2)	
N4	1.1199 (2)	0.64457 (11)	0.75251 (8)	0.0155 (2)	
C1	0.7751 (2)	0.58432 (11)	0.92934 (8)	0.0128 (2)	
C2	0.9508 (2)	0.59135 (11)	0.79616 (8)	0.0121 (2)	
C3	0.7835 (2)	0.49584 (11)	0.75646 (8)	0.0139 (2)	
H3A	0.7827	0.4627	0.6968	0.017*	
C4	0.6220 (2)	0.45442 (11)	0.81056 (8)	0.0138 (2)	
O1	0.1038 (2)	0.65131 (9)	0.56049 (7)	0.0197 (2)	
O2	0.2393 (2)	0.68272 (9)	0.42877 (7)	0.0196 (2)	
C5	0.2377 (2)	0.62262 (11)	0.50395 (8)	0.0145 (2)	
C6	0.4095 (2)	0.51141 (11)	0.52358 (8)	0.0148 (2)	
H6A	0.3894	0.4547	0.5715	0.018*	
H1	1.054 (4)	0.688 (2)	0.9060 (19)	0.054 (8)*	
H2	0.874 (4)	0.685 (2)	1.0338 (16)	0.031 (6)*	
H3	0.662 (4)	0.597 (2)	1.0383 (16)	0.035 (6)*	
H4	1.118 (4)	0.629 (2)	0.7009 (16)	0.024 (5)*	
H5	1.208 (4)	0.697 (2)	0.7770 (15)	0.025 (5)*	

### Atomic displacement parameters (Å^2^)

**Table:**

	*U*^11^	*U*^22^	*U*^33^	*U*^12^	*U*^13^	*U*^23^
Cl1	0.01810 (17)	0.01552 (16)	0.02516 (18)	−0.00602 (9)	0.00670 (12)	−0.00792 (10)
N1	0.0156 (4)	0.0128 (4)	0.0160 (5)	−0.0033 (3)	0.0054 (3)	−0.0023 (3)
N2	0.0156 (4)	0.0097 (4)	0.0130 (4)	−0.0027 (3)	0.0050 (3)	−0.0008 (3)
N3	0.0212 (5)	0.0169 (5)	0.0153 (5)	−0.0072 (4)	0.0082 (4)	−0.0037 (4)
N4	0.0205 (5)	0.0133 (4)	0.0138 (4)	−0.0028 (4)	0.0063 (4)	−0.0010 (4)
C1	0.0144 (5)	0.0103 (5)	0.0145 (5)	−0.0017 (4)	0.0047 (4)	0.0002 (4)
C2	0.0139 (5)	0.0094 (4)	0.0135 (5)	0.0008 (3)	0.0041 (4)	0.0004 (3)
C3	0.0155 (5)	0.0114 (5)	0.0154 (5)	−0.0015 (4)	0.0047 (4)	−0.0020 (4)
C4	0.0141 (5)	0.0101 (4)	0.0178 (5)	−0.0014 (4)	0.0040 (4)	−0.0022 (4)
O1	0.0235 (5)	0.0206 (5)	0.0174 (4)	0.0087 (3)	0.0096 (4)	0.0032 (3)
O2	0.0270 (5)	0.0178 (4)	0.0159 (4)	0.0112 (4)	0.0089 (4)	0.0055 (3)
C5	0.0167 (5)	0.0132 (5)	0.0138 (5)	0.0039 (4)	0.0039 (4)	0.0004 (4)
C6	0.0179 (5)	0.0126 (5)	0.0143 (5)	0.0049 (4)	0.0039 (4)	0.0023 (4)

### Geometric parameters (Å, º)

**Table:**

Cl1—C4	1.7385 (12)	N4—H4	0.78 (2)
N1—C4	1.3305 (15)	N4—H5	0.77 (2)
N1—C1	1.3474 (15)	C2—C3	1.4076 (16)
N2—C2	1.3529 (15)	C3—C4	1.3695 (16)
N2—C1	1.3592 (14)	C3—H3A	0.9500
N2—H1	0.862 (10)	O1—C5	1.2482 (14)
N3—C1	1.3273 (15)	O2—C5	1.2824 (14)
N3—H2	0.81 (2)	C5—C6	1.4919 (16)
N3—H3	0.85 (2)	C6—C6^i^	1.334 (2)
N4—C2	1.3444 (15)	C6—H6A	0.9500
			
C4—N1—C1	114.99 (10)	N4—C2—C3	122.99 (11)
C2—N2—C1	120.34 (10)	N2—C2—C3	119.54 (10)
C2—N2—H1	118 (2)	C4—C3—C2	114.80 (10)
C1—N2—H1	122 (2)	C4—C3—H3A	122.6
C1—N3—H2	119.5 (17)	C2—C3—H3A	122.6
C1—N3—H3	113.3 (16)	N1—C4—C3	127.36 (11)
H2—N3—H3	127 (2)	N1—C4—Cl1	114.05 (9)
C2—N4—H4	120.1 (17)	C3—C4—Cl1	118.59 (9)
C2—N4—H5	119.4 (16)	O1—C5—O2	124.44 (11)
H4—N4—H5	120 (2)	O1—C5—C6	118.95 (11)
N3—C1—N1	119.22 (10)	O2—C5—C6	116.61 (10)
N3—C1—N2	117.81 (11)	C6^i^—C6—C5	122.53 (14)
N1—C1—N2	122.97 (10)	C6^i^—C6—H6A	118.7
N4—C2—N2	117.47 (11)	C5—C6—H6A	118.7
			
C4—N1—C1—N3	−179.71 (11)	N2—C2—C3—C4	0.33 (17)
C4—N1—C1—N2	−0.05 (17)	C1—N1—C4—C3	0.85 (19)
C2—N2—C1—N3	179.15 (11)	C1—N1—C4—Cl1	−178.65 (9)
C2—N2—C1—N1	−0.51 (18)	C2—C3—C4—N1	−0.99 (19)
C1—N2—C2—N4	179.70 (11)	C2—C3—C4—Cl1	178.50 (9)
C1—N2—C2—C3	0.34 (17)	O1—C5—C6—C6^i^	−167.18 (16)
N4—C2—C3—C4	−178.98 (11)	O2—C5—C6—C6^i^	12.7 (2)

Symmetry code: (i) −*x*+1, −*y*+1, −*z*+1.

### Hydrogen-bond geometry (Å, º)

**Table:**

*D*—H···*A*	*D*—H	H···*A*	*D*···*A*	*D*—H···*A*
N2—H1···O2^ii^	0.86 (1)	1.69 (1)	2.5281 (14)	165 (3)
N3—H2···O1^ii^	0.81 (2)	2.12 (2)	2.9233 (15)	168 (2)
N3—H3···N1^iii^	0.85 (2)	2.15 (2)	3.0014 (16)	176 (2)
N4—H4···O1^iv^	0.78 (2)	2.08 (2)	2.8307 (16)	161 (2)
N4—H5···Cl1^v^	0.77 (2)	2.78 (2)	3.3671 (13)	135.0 (19)
N4—H5···O2^ii^	0.77 (2)	2.56 (2)	3.1458 (15)	134.2 (19)
C3—H3*A*···O2^i^	0.95	2.39	3.3085 (16)	162

Symmetry codes: (i) −*x*+1, −*y*+1, −*z*+1; (ii) *x*+1, −*y*+3/2, *z*+1/2; (iii) −*x*+1, −*y*+1, −*z*+2; (iv) *x*+1, *y*, *z*; (v) −*x*+2, *y*+1/2, −*z*+3/2.
